# Phage-Resistant Bacteria Reveal a Role for Potassium in Root Colonization

**DOI:** 10.1128/mBio.01403-21

**Published:** 2021-08-17

**Authors:** Elhanan Tzipilevich, Philip N. Benfey

**Affiliations:** a Howard Hughes Medical Institute, Duke University, Durham, North Carolina, USA; b Department of Biology, Duke University, Durham, North Carolina, USA; Universidade de Sao Paulo

**Keywords:** *Bacillus subtilis*, bacteriophage, evolution, biofilms, plant-microbe interactions

## Abstract

Bacteriophage predation is an important factor in bacterial community dynamics and evolution. Phage-bacterium interaction has mainly been studied in lab cultures, while dynamics in natural habitats, and especially in the plant root niche, are underexplored. To better understand this process, we characterized infection of the soil bacterium Bacillus subtilis NCBI 3610 by the lytic phage SPO1 during growth in LB medium and compared it to root colonization. Resistance *in vitro* was primarily through modification of the phage receptor. However, this type of resistance reduced the ability to colonize the root. From a line that survived phage infection while retaining the ability to colonize the root, we identified a new phage resistance mechanism involving potassium (K^+^) ion influx modulation and enhanced biofilm formation. Furthermore, we show that potassium serves as a stimulator of root colonization among diverse growth-promoting bacilli species, with implications for plant health.

## INTRODUCTION

Plant roots are associated with diverse bacteria in the soil (1), which can affect many aspects of plant life, including root architecture (2), nutrient acquisition (3), and disease state (2, 4). The root rhizosphere, i.e., the area close to the root surface, is enriched with specific bacterial taxa in comparison to that in bulk soil. This unique microbial composition is determined by the soil surrounding the plant root and its preexisting bacterial diversity (5, 6) and plant genotype (7) as well as the interaction with other bacteria (8) and with phage (9). Although a large body of research has been conducted to characterize each of these factors, the whole picture, and especially the role of phage in bacterial root colonization, is far from complete.

Phage are viruses that infect and kill bacteria (10, 11). As bacterial predators, they influence bacterial community dynamics through elimination of their sensitive hosts. Bacteria, in turn, respond by rapid evolution of phage resistance (12). In addition to influencing bacterial interaction with phage, newly acquired resistance (10, 13) can also cause changes in colony morphology (14), genome-wide mutation rate (15), and lateral spread of genetic material (16). In recent years, insights have been gained into how phage-bacterium interactions affect bacterial growth in ocean (17) and mammalian gut ecosystems (18). However, little is known about the effects of phage-bacterium interactions in other ecosystems, especially in soil and the root rhizosphere (19).

To address this question, we utilized Bacillus subtilis strain NCBI 3610 (henceforth, B. subtilis) (20) and its cognate lytic phage SPO1 (21) to explore their interaction during root colonization of the plant model system Arabidopsis thaliana. B. subtilis is a Gram-positive spore-forming bacterium isolated from soil and is able to colonize plant roots (22). Root colonization by B. subtilis is mediated by formation of a biofilm on the root (23). Biofilms are bacterial communities encased in an extracellular matrix. The B. subtilis matrix is mainly composed of sugar polymers, encoded by the *eps* operon, and protein fibers encoded by the *tapA-sipW-tasA* operon (24). B. subtilis defective in biofilm formation exhibits severe defects in root colonization (23). The phage SPO1 is a lytic phage, representing a large and diverse group of *Myoviridae* bacteriophages, harboring a long contractile tail. SPO1 phage exhibits a complex infection cycle, involving the subversion of the host transcription machinery for its own use (25). SPO1 utilizes the wall teichoic acid polymers (WTAs) as receptors to invade B. subtilis cells (26). WTAs are long sugar polymers that are incorporated into the cell wall and membrane of B. subtilis and other Gram-positive bacteria (27). SPO1 binds these polymers when they are decorated by glucose moieties (gWTAs). Comparison of phage-bacterium evolution upon infection in LB medium and during root colonization revealed that phage infection *in vitro* and *in planta* exhibits different evolutionary trajectories. Characterization of bacteria resistant to phage infection during root colonization led to the identification of a novel phage resistance mechanism through modulation of potassium (K^+^) ion influx and enhanced biofilm formation. Furthermore, we show that potassium serves as a stimulator of root colonization among diverse bacilli species.

## RESULTS

### Loss of phage receptor results in a fitness cost for root colonization.

**FIG 1:**
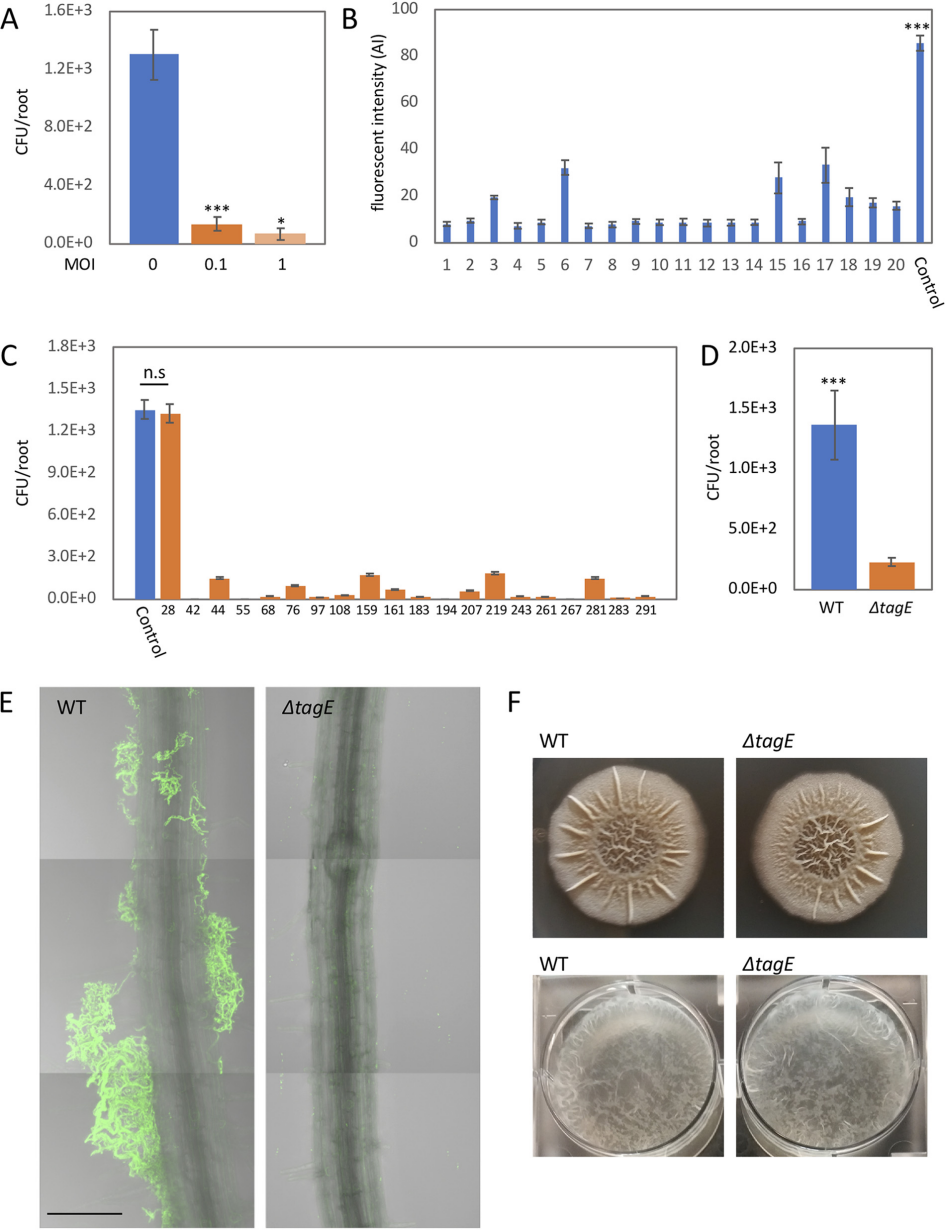
SPO1 phage receptor is necessary for bacterial root adhesion. (A) Seedlings were inoculated with B. subtilis 3610 together with SPO1 phage, at either a high (phage/bacteria, 1:1) or low (phage/bacteria, 1:10) multiplicity of infection (MOI) or with no phage (MOI of 0) for 48 h on agar plates, and the number of colonizing bacteria was counted. Shown are averages and standard deviations (SDs) from 2 independent experiments with an *n* of ≥3 for each (*n* throughout the paper is the number of roots sampled [biological replicates] with 3 technical replicates from each root). *, *P* < 0.05; ***, *P* < 0.005. (B) Twenty randomly selected bacterial colonies that survived SPO1 infection were stained with ConA_488_ and observed under a compound microscope. Shown are average and SD fluorescent intensity values of 10 bacteria from each of the colonies. ***, *P* < 0.005. (C) Twenty isolated SPO1 resistant bacteria were inoculated on seedlings and grown for 48 h, and the number of colonizing bacteria were counted. Shown are averages and SDs with an *n* of ≥3. (D) Seedlings were inoculated with either WT or Δ*tagE* bacteria for 48 h, and the number of colonizing bacteria was counted. Shown are averages and SDs from 2 independent experiments with an *n* of ≥3 for each. ***, *P* < 0.005. (E) Seedlings were inoculated with either WT or Δ*tagE* bacteria expressing green fluorescent protein (GFP) (amyE::P*_rrnE_*-gfp) for 48 h on agar plates. Shown are representative overlay ×200 magnification confocal images of differential interference contrast (DIC) (root) and GFP fluorescence (bacteria). Scale bar, 50 μm. (F) WT and Δ*tagE* bacteria were inoculated onto MSgg agar plates (top) or liquid medium (bottom). Shown are representative biofilm images.

**FIG 2:**
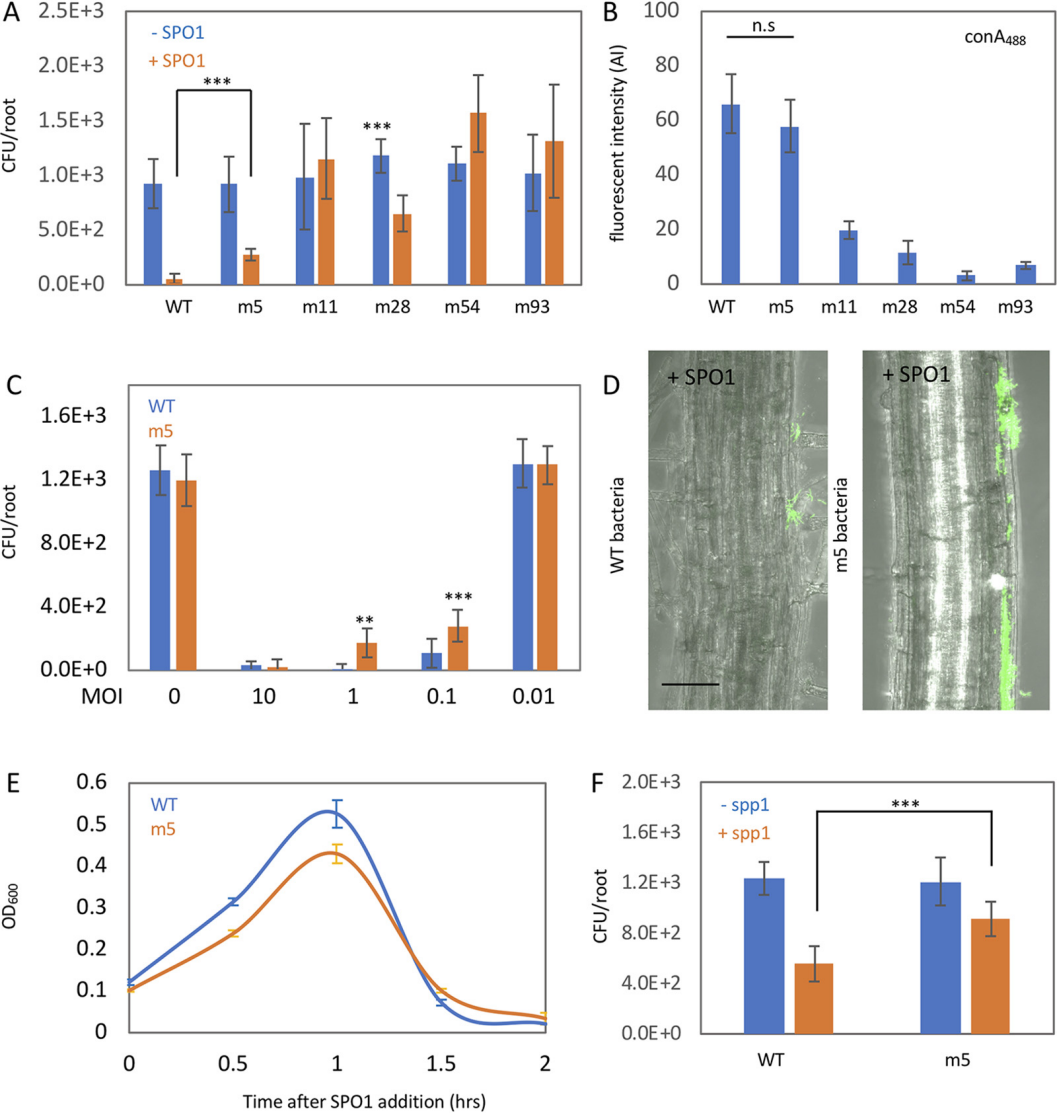
A novel SPO1 resistance mechanism. (A) Seedlings were inoculated with the indicated bacterial strains, with or without SPO1 addition, for 48 h on agar plates, and the number of colonizing bacteria was counted. Shown are averages and SDs from 2 independent experiments with an *n* of ≥3 for each. ***, *P* < 0.005. (B) Bacterial strains were stained with ConA_488_ and observed under the microscope. Shown are average and SD of fluorescence intensity values of 10 bacteria from each colony. n.s., not significant. (C) Seedlings were inoculated with either WT or m5 bacteria, with the indicated phage MOI, for 48 h on agar plates, and the number of colonizing bacteria was counted. Shown are averages and SDs from 2 independent experiments with an *n* of ≥3 for each. **, *P* < 0.01; ***, *P* < 0.005. (D) Seedlings were inoculated with either WT or m5 bacteria expressing GFP (amyE::P*_rrnE_*-gfp), in the presence of SPO1, for 48 h on agar plates. Shown are representative overlay ×200 magnification confocal images of DIC (root) and GFP fluorescence (bacteria). Scale bar, 20 μm. (E) WT and m5 bacteria were infected with SPO1 in LB medium, and OD_600_ was followed. Shown are averages and SDs, *n* = 3. (F) Seedlings were inoculated with either WT or m5 bacteria, with or without SPP1 phages, for 48 h on agar plates, and the number of colonizing bacteria was counted. Shown are averages and SDs from 2 independent experiments with an *n* of ≥3 for each. ***, *P* < 0.005.

To explore phage-bacterium interactions during root colonization, we inoculated the roots of *A. thaliana* with B. subtilis 3610 bacteria together with SPO1 phage at either a high (phage/bacteria, 1:1), or low (phage/bacteria, 1:10) multiplicity of infection (MOI). Measuring bacterial colonization (CFU) after 48 h revealed a reduction in root colonization of ∼95% at an MOI of 1 and an ∼90% reduction at an MOI of 0.1 (Fig. 1A), indicating that SPO1 can efficiently infect and kill its host bacteria during root colonization. Significant levels of PFU were recovered from the root after SPO1 infection (5.56 × 10^4^ ± 4.67 × 10^4^, for MOI of 1, *n* = 6). However, no increase in the amount of the phage was observed in comparison to the input (PFU = 10^6^), probably reflecting the fact that phage cannot adhere to the root without the presence of the host bacteria. Selection pressure by phage drives bacterial evolution of resistance mechanisms (28). To explore the evolution of phage resistance mechanisms *in vitro* versus those *in planta*, we isolated 300 bacteria (screen 1) that survived phage infection on the root and restreaked them on agar plates containing SPO1 phage, (maximum of 10 bacteria from each root). We found 20 *in planta* survivors that became resistant to phage infection *in vitro*. On the other hand, 100% of 70 bacteria isolated after surviving phage infection in LB medium (taken from 3 independent LB plates) became immune to further infection. Twenty randomly selected *in vitro* survivors became phage resistant through loss of the phage receptor, as judged by a lack of staining with concanavalin A, Alexa Fluor 488 conjugate (ConA_488_), a lectin that binds specifically glycosylated WTA (gWTA) (Fig. 1B) (see also reference 26). Of the 20 SPO1-resistant bacteria isolated from the plant all but one (m28) had lost their ability to recolonize the root (Fig. 1C). Nineteen of these isolates concomitantly lost their ConA_488_ staining (see Fig. S1A in the supplemental material). One of the isolates, m28, was completely phage resistant *in vitro* when infected in liquid culture (Fig. S1B) but still exhibited faint ConA_488_ staining (Fig. S1A and C) and partial sensitivity when infected on roots (Fig. 2A). The correlation between SPO1 sensitivity and root colonization ability suggests that gWTA is important for efficient root colonization. To test this hypothesis, we inoculated roots with Δ*tagE* bacteria, which lack gWTA (29), and found a significant reduction in root colonization by CFU measurement and fluorescent bacteria (Fig. 1D and E). Because biofilm formation has been shown to be necessary for root colonization by B. subtilis (30), we tested the ability of Δ*tagE* bacteria to form a biofilm *in vitro*. Δ*tagE* cells exhibit normal biofilm formation both on agar plates and in liquid medium (Fig. 1F). Of note, gWTA is required for nasal epithelium colonization by Staphylococcus aureus (31). Our results suggest that gWTA in B. subtilis is similarly important for plant surface adhesion, irrespective of biofilm formation.

10.1128/mBio.01403-21.1 FIG S1 SPO1 resistance profile of phage survival isolates. (A) SPO1-resistant bacteria isolated from plants were stained with ConA_488_ and observed under the microscope. Shown are average and SD fluorescent intensity values from 10 bacteria of each of the surviving colonies. (B) The indicated bacterial strains were infected with SPO1 in LB medium, and OD_600_ was followed. Shown are averages and SDs with an *n* of 3. (C) The indicated bacterial strains were stained with ConA_488_. Shown are representative DIC (left) and fluorescence from ConA_488_ (right) images from each strain. Download FIG S1, TIF file, 2.3 MB. Copyright © 2021 Tzipilevich and Benfey. 2021 Tzipilevich and Benfey. https://creativecommons.org/licenses/by/4.0/ This content is distributed under the terms of the Creative Commons Attribution 4.0 International license.

### Bacteria evolved a novel phage resistance mechanism.

Our results indicate that loss of the phage receptor results in a fitness cost to bacteria during root colonization. To identify alternative pathways utilized by bacteria to resist phage infection during root colonization, we utilized the bacteria that survived phage infection on the root (100 of the 300 bacteria from screen 1) and infected them with SPO1, this time during root colonization (screen 2). Most of the bacteria exhibited phage sensitivity similar to that of the parental bacteria. However, of 100 bacterial strains tested, we found 4 that survived SPO1 infection on roots better than wild-type (WT) cells (Fig. 2A). To explore the mechanism of bacterial survival, we sequenced the genomes of m5, m11, m54, and m93, the 4 bacteria isolated during screen 2, along with m28 isolated from screen 1. Table 1 presents the mutations in each of the bacteria.

**TABLE 1:** Bacterial strains

Bacterial strain	Mutation
m5	Missense mutation in KtrC P189T
m11	Mutations in YwbO promoter
m28	Missense mutation GtaB Y170S
m54	Missense mutations in YwrK T334S and CdaA F97L
m93	Frame shift in *tagE* T1013 leading to premature stop codon

m28 and m93 harbor mutations in *gtaB* and *tagE*, genes involved in the WTA glycosylation pathway (27). Interestingly, m11 and m54 do not have mutations in genes previously implicated in WTA glycosylation but nonetheless exhibited complete (m54) and partial (m11) loss of ConA_488_ staining (Fig. 2B and Fig. S1C). m11, m54, and m93 exhibited phage resistance *in vitro* (Fig. S1B).

One mutant, m5, exhibited enhanced phage resistance when infected *in planta* at a medium MOI (1 to 0.1) (Fig. 2C, CFU, and Fig. 2D, fluorescent bacteria), phage sensitivity *in vitro*, and ConA_488_ staining similar to that of WT (Fig. 2B and E and Fig. S2B), indicating that it harbors a novel phage resistance mechanism. The m5 mutant also exhibited enhanced resistance to infection by SPP1, a lytic phage from a different bacteriophage family (*Siphoviridae*) (Fig. 2F). SPP1 utilizes YueB, a membrane protein, as a receptor. However, gWTA also plays an important role for SPP1 infection as well (32). Nevertheless, m5 has a resistance mechanism that is not phage-family specific. m5 harbors a single point mutation in the *ktrC* gene, encoding a low-affinity potassium channel. This missense mutation changes proline 189 into threonine (KtrC P189T) and resides in a conserved RCK domain, known to bind the regulatory molecule ci-di-AMP (33, 34). ci-di-AMP negatively regulates potassium uptake (35). We hypothesized that KtrC P189T is a gain of function mutation, affecting the regulation of potassium uptake.

10.1128/mBio.01403-21.2 FIG S2 Potassium modulates B. subtilis root colonization and phage resistance. (A) Seedlings were inoculated with WT bacteria and grown for 48 h on 0.25× MS agar plates in the absence or presence of 5 mM of the indicated salts, and the number of colonizing bacteria was counted. Shown are averages and SDs from 2 independent experiments with an *n* of ≥3 for each. ***, *P* < 0.005. (B) Seedlings were inoculated with Δ*kinC* bacteria, with or without SPO1 addition, and grown for 48 h on 0.25× MS agar plates in the presence or absence of 5 mM KCl; the number of colonizing bacteria was counted. Shown are averages and SDs from 2 independent experiments with an *n* of ≥3 for each. (C) Seedlings were inoculated with the indicated bacterial strains and grown for 48 h on 0.25× MS agar plates, in the absence or presence of 5 mM KCl, and the number of colonizing bacteria was counted. Shown are averages and SDs from 2 independent experiments with an *n* of ≥3 for each. ***, *P* < 0.005. Download FIG S2, TIF file, 2.3 MB. Copyright © 2021 Tzipilevich and Benfey. 2021 Tzipilevich and Benfey. https://creativecommons.org/licenses/by/4.0/ This content is distributed under the terms of the Creative Commons Attribution 4.0 International license.

### Potassium enhances root colonization and phage resistance through modulation of biofilm formation.

**FIG 3:**
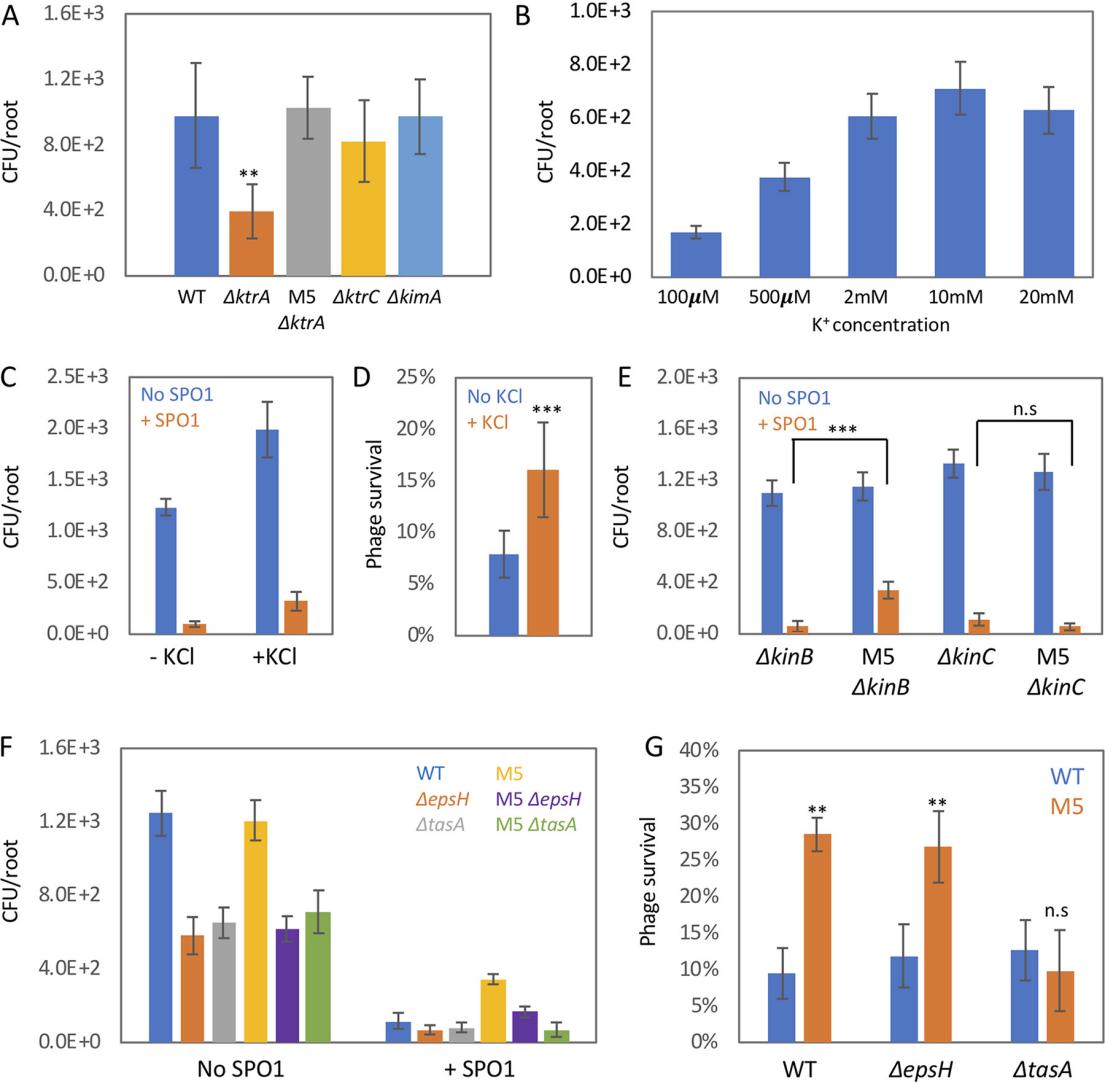
Potassium modulates B. subtilis root colonization and phage resistance through biofilm formation. (A) Seedlings were inoculated with the indicated bacterial strains for 48 h on agar plates, and the number of colonizing bacteria was counted. Shown are averages and SDs from 2 independent experiments with an *n* of ≥3 for each. **, *P* < 0.01. (B) Seedlings were inoculated with WT B. subtilis on MS agar plates supplemented with the indicated potassium concentration for 48 h, and the number of colonizing bacteria was counted. Shown are averages and SDs from 2 independent experiments with an *n* of ≥3 for each. (C and D) Seedlings were inoculated with WT B. subtilis, with or without SPO1 addition, for 48 h on 0.25× MS agar plates in the presence or absence of 5 mM KCl, and the number of colonizing bacteria was counted. Shown are averages and SDs of CFU values (C) and the percentages of SPO1 survival (D), from 2 independent experiments with an *n* of ≥3 for each. ***, *P* < 0.005. (E) Seedlings were inoculated with the indicated bacterial strains, with or without SPO1 addition, for 48 h on agar plates, and the number of colonizing bacteria was counted. Shown are averages and SDs from 2 independent experiments with an *n* of ≥3 for each. ***, *P* < 0.005. (F and G) Seedlings were inoculated with the indicated bacterial strains, with or without SPO1 addition, for 48 h on agar plates in the presence or absence of 5 mM KCl, and the number of colonizing bacteria was counted. Shown are average and SD CFU values (F) and percentages of SPO1 survival (G) from 2 independent experiments with an *n* of ≥3 for each, **, *P* < 0.01.

Several potassium channels are encoded in the B. subtilis genome: *ktrAB* and *kimA* encode high-affinity channels (36, 37), while the *ktrCD* operon encodes a low-affinity channel (36). To understand the effect of the KtrC P189T mutation and potassium uptake on bacterial root colonization, we examined the colonization efficiency of bacteria lacking these channels. While Δ*ktrC* and Δ*kimA* mutants colonized the root with similar efficiency to WT bacteria (Fig. 3A), Δ*ktrA* bacteria exhibited reduced root colonization (Fig. 3A). The KtrC P189T mutation is able to restore the colonization of Δ*ktrA* bacteria (Fig. 3A, M5 Δ*ktrA*), suggesting that this mutation enhances potassium uptake through the KtrC channel. Growing plants on media with different levels of potassium revealed a positive correlation between potassium concentration and root colonization by WT bacteria (Fig. 3B), with the effect plateauing at 10 mM. Similarly, root colonization on 0.25 Murashige and Skoog (MS) plates, with addition of 5 mM potassium (KCl) (equal to total of ∼10 mM) indicated a positive effect of potassium on root colonization (Fig. 3C). Stimulation of root colonization was specific to potassium ions, as neither sodium nor nitrogen had a similar effect (Fig. S2A). Simply adding extra potassium to the plant growth medium was sufficient to enhance survival of WT cells upon phage infection (Fig. 3C and D), reinforcing the idea that the KtrC P189T mutation affects phage infection through increased potassium uptake.

It was previously shown that B. subtilis bacteria sense potassium influx through the cell membrane, utilizing KinC and KinB protein kinases to induce biofilm formation genes and sliding motility, respectively (38, 39). Both biofilm formation and sliding motility play important roles during root colonization (23, 40). We found that m5 Δ*kinC* bacteria, but not m5 Δ*kinB* bacteria, lost SPO1 resistance (Fig. 3E). Addition of potassium to the plant growth medium failed to stimulate phage resistance for Δ*kinC* bacteria (Fig. S2C). Thus, we conclude that KinC is involved in the effect of potassium on root colonization and phage resistance.

KinC activation induces a phosphorylation cascade that culminates in induction of biofilm matrix genes (38). Disruption of the biofilm matrix operons *epsA-O* and *tapA-sipW-tasA* abolished the effect of potassium on root colonization (Fig. S2C). Interestingly, disruption of the *tapA-sipW-tasA* operon, but not *epsA-O*, reduced the phage resistance phenotype of m5 cells (Fig. 3F and G), suggesting that an increase in the protein fiber component was responsible for the phage resistance effect of increased potassium influx. Thus, our analysis revealed a novel phage adaptation mechanism that works through enhanced potassium influx by stimulating biofilm matrix formation.

**FIG 4:**
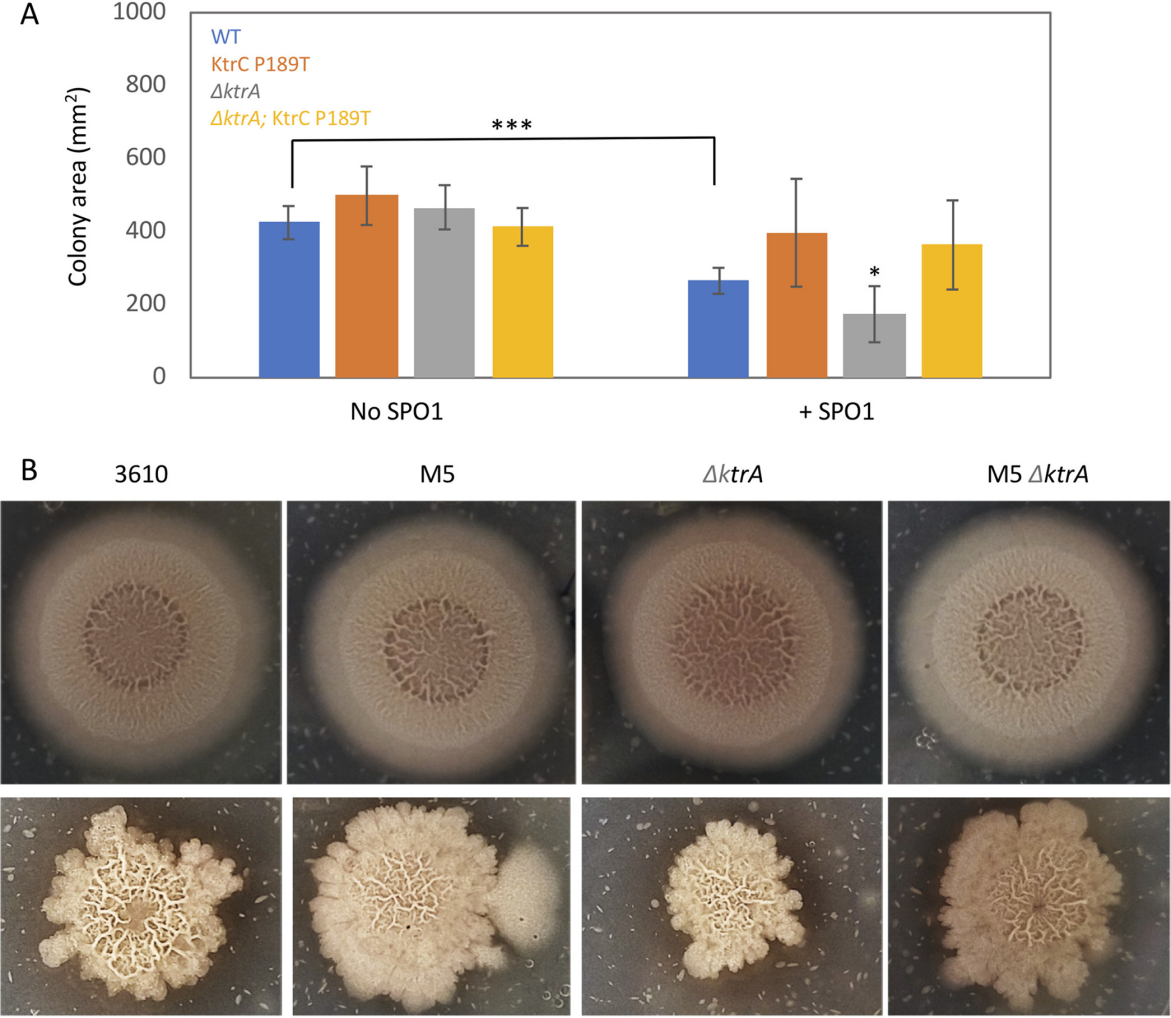
Potassium modulates phage resistance during biofilm formation *in vitro*. (A) The indicated bacterial strains were inoculated onto MSgg agar plates with or without SPO1 addition for 48 h, and colony area was measured. Shown are averages and SDs from an *n* of 6. *, *P* < 0.05; ***, *P* < 0.005. (B) Shown are representative images for the experiment described for panel A with (bottom) or without (top) SPO1 addition.

To further characterize the role of biofilm matrix formation in phage resistance, we monitored SPO1 infection during biofilm formation *in vitro* (20). Measurement of biofilm diameter on MSgg agar plates revealed significant reduction in colony diameter on plates containing SPO1 phage (Fig. 4A). Of note, Δ*ktrA* cells, although able to form normal biofilm in the absence of phage, exhibited a significant decrease in biofilm diameter in the presence of phage in comparison to that of WT infected cells (Fig. 4A and B). Similar to what was observed in plants, the KtrC P189T mutation was able to compensate for the increased sensitivity of Δ*ktrA* cells (Fig. 4A and B). Thus, our *in vitro* analysis provides further support for the hypothesis that potassium influx modulates biofilm formation to enhance phage resistance. Equivalent results were obtained for pellicle biofilm formation on liquid MSgg medium (Fig. S3), where enhanced potassium influx in m5 bacteria, or addition of extra potassium to the medium of WT bacteria, increased the chance of surviving SPO1 infection, while decreased influx, due to the Δ*ktrA* mutation, increased phage sensitivity.

10.1128/mBio.01403-21.3 FIG S3 Potassium modulates phage resistance during pellicle biofilm formation. (A) The indicated bacterial strains were inoculated in MSgg medium with or without SPO1 addition for 48 h, and the number of cultures exhibiting partial SPO1 survival (see panel C) were counted. Shown are the percentages of the cultures surviving SPO1, *n* ≥ 30. ***, *P* < 0.005. (B) WT bacteria were inoculated into MSgg medium with or without 5 mM KCl and infected with SPO1 for 48 h, as described for panel A. Shown are the percentages of the cultures surviving SPO1, *n* ≥ 30. ***, *P* < 0.005. (C) Shown are representative images for the experiment described in panels A and B, for an uninfected culture (1), a culture undergoing complete phage lysis (2), and a culture counted as partially surviving phage infection (3). Download FIG S3, TIF file, 2.3 MB. Copyright © 2021 Tzipilevich and Benfey. 2021 Tzipilevich and Benfey. https://creativecommons.org/licenses/by/4.0/ This content is distributed under the terms of the Creative Commons Attribution 4.0 International license.

### Potassium enhances root colonization by diverse bacilli species.

**FIG 5:**
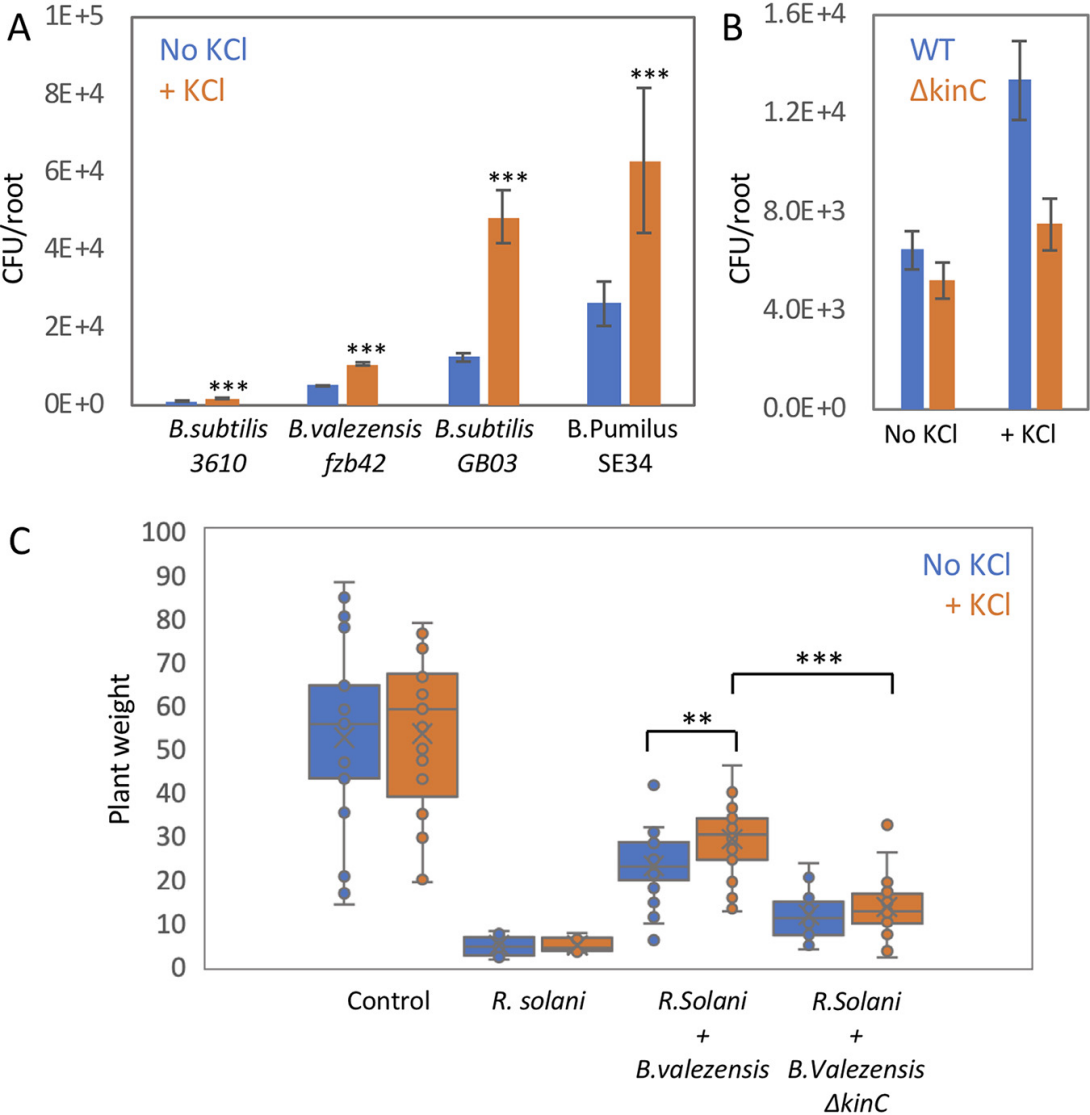
Potassium modulates root colonization by diverse bacillus species. (A) Seedlings were inoculated with the indicated bacterial species for 48 h on 0.25× MS agar plates in the presence or absence of 5 mM KCl, and the number of colonizing bacteria was counted. Shown are averages and SDs from 2 independent experiments with an *n* of ≥3 for each. ***, *P* < 0.005. (B) Seedlings were inoculated with either WT or Δ*kinC B. velezensis* FZB42 bacteria for 48 h on 0.25× MS agar plates in the presence or absence of 5 mM KCl, and the number of colonizing bacteria was counted. Shown are averages and SDs from 2 independent experiments with and *n* of ≥3 for each. (C) Seedlings were inoculated with either WT or Δ*kinC B. velezensis* FZB42 bacteria, for 48 h on 0.25× MS agar plates in the presence or absence of 5 mM KCl. Next, plates were inoculated with R. solani and incubated for an additional 7 days, and plant weight was measured. Untreated plants (neither bacteria nor fungi) were used as control. Shown are averages and SDs, *n* ≥ 20. **, *P* < 0.01; ***, *P* < 0.005.

To determine if potassium is able to enhance colonization by other bacilli species, we analyzed its effect on Bacillus velezensis FZB42, a plant-associated bacterium (41), shown to enhance plant health through growth stimulation and pathogen inhibition. We found that potassium enhances root colonization of *B. velezensis* FZB42 (Fig. 5A), and this phenomenon was abolished in *B. velezensis* FZB42 Δ*kinC* cells (Fig. 5B). A similar phenomenon was observed for B. subtilis GB03 and B. pumilus SE34, two other plant growth-promoting bacteria (Fig. 5A). Thus, potassium serves as a wide-spread signal, stimulating root colonization by diverse bacilli species. *B. velezensis* FZB42 inhibits the growth of the plant fungal pathogen Rhizoctonia solani (42), raising the possibility that simply adding potassium to the growth medium could enhance *B. velezensis* FZB42 colonization and fungal protection. Indeed, potassium enhanced plant growth when inoculated with Rhizoctonia solani in the presence of *B. velezensis* FZB42 in comparison to growth of plants inoculated with fungi but no bacteria (Fig. 5C), and this effect of plant protection was abolished in Δ*kinC* cells, which are unable to sense potassium influx (Fig. 5C).

## DISCUSSION

Phage infection has been extensively characterized in lab cultures. Recent work revealed that phage-bacterium interactions in natural environments can differ significantly from those observed in the lab (43) due to the fitness constraints encountered by bacteria in their natural environment. This can impede the evolution of phage receptors, the main resistance pathway observed in the lab (44). We found that gWTA, the SPO1 phage receptor, is essential for root adhesion; thus, receptor modification has a fitness cost in the root environment. Of note, it has been shown that gWTA is essential for adhesion of Staphylococcus aureus to the nasal epithelium (31), while gWTA also serves as phage receptor for several S. aureus-infecting phages (45). It will be interesting to determine if phage-bacterium evolution is constrained in this niche as well.

**FIG 6:**
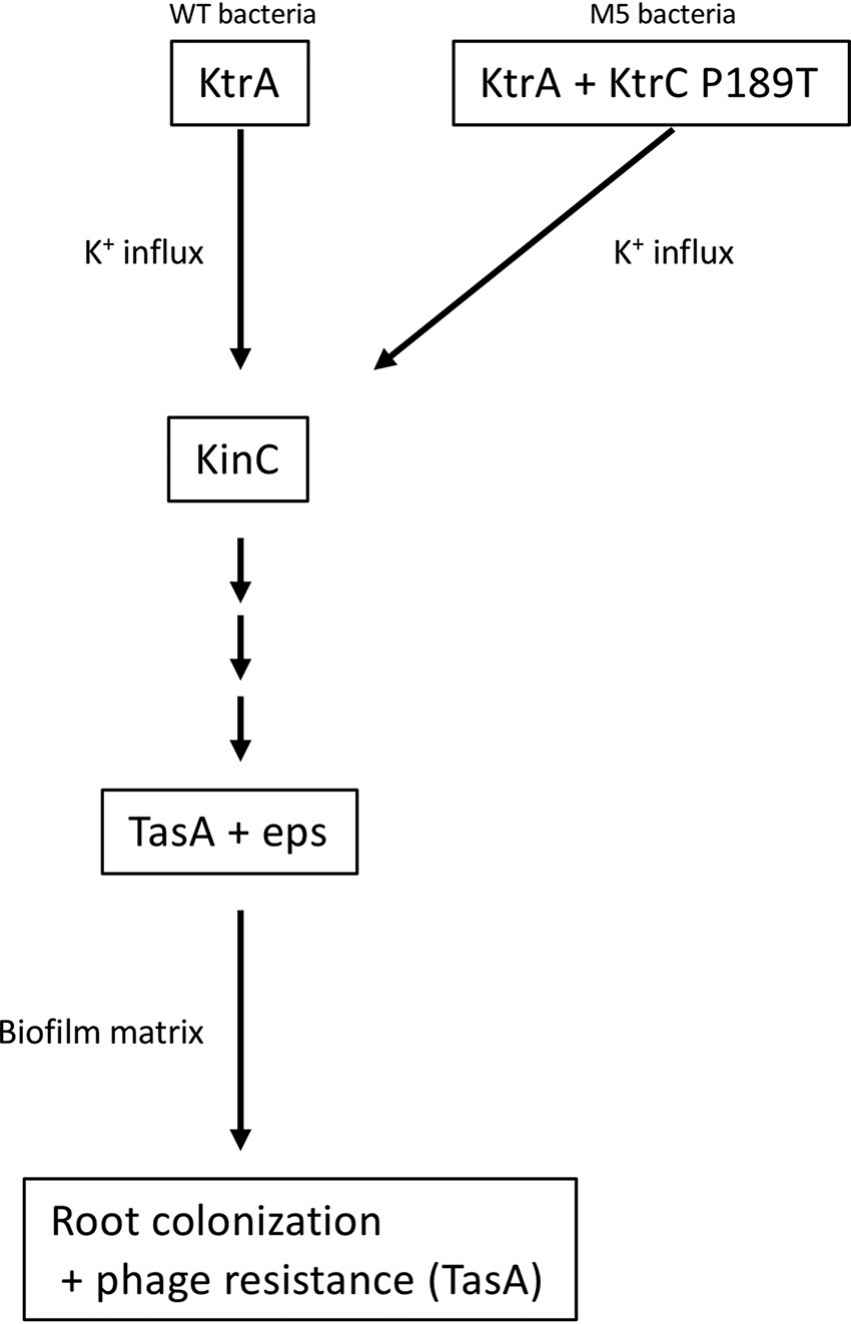
A model describing the effect potassium on root colonization and phage resistance.

While evolution of receptor modification is not favored, we found that bacteria in the root adapt to phage infection through evolution of enhanced biofilm formation. Consistent with previous *in vitro* work (38), we show that during plant colonization, altering potassium efflux through KtrCD channels enhances B. subtilis biofilm formation and thus promotes survival upon phage infection (Fig. 6). Our results indicate that protein fiber density, encoded by the *tapA-sipW-tasA* operon, is the main determinant of SPO1 resistance. Interestingly, it has been shown that curli protein fibers are important for T7 resistance in a biofilm of Escherichia coli bacteria (46). Phage-bacterium interaction in biofilm is an emerging area of research (47). Biofilms are an important mode of life for bacteria colonizing natural environments, such as plant roots, as well as for pathogenic bacteria (48). Thus, in order to understand bacterial community dynamics in nature, and to deal with biofilm pathogenicity, it is important to explore the interaction of phage and bacteria in this specific niche.

Phage utilize bacterial appendages decorating the cell surface as receptors. Appendages such as flagella, type IV pili, exopolysaccharides, and transporters of nutrients, are important for bacterial survival in the natural environment and specifically, in the plant niche (9). Evolution of phage resistance has been associated with reduced host fitness on plants. An example is phage utilizing type IV pili (49), where the evolved mutant bacteria growly similar to the parental strain in planktonic culture but suffer from reduced biofilm formation and plant leaf colonization. Thus, it is important to explore phage-bacterium interactions in their natural context to gain insight into their coevolutionary dynamics.

Our genetic screen opens the way to discovering new phage resistance strategies that are not accessible during *in vitro* growth. An interesting future direction derives from the observation that among *in planta* phage-resistant bacteria, we found 4 of 100 with genetically inherited enhanced phage resistance. This suggests that 96% of the bacteria survived infection in a nongenetic manner. It would be interesting to determine how nongenetic mechanisms enable bacteria to survive phage infection. One possibility is by colonizing root niches that are less accessible to phage, a strategy that was recently demonstrated for phage infection of bacteria colonizing the gut ecosystem (50).

Potassium is an essential macronutrient for bacteria and plants. Unlike nitrogen and phosphate, it is not incorporated into cellular macromolecules but remains a soluble ion (51). Potassium is involved in the regulation of many processes, including osmotic adaptation, membrane potential, phloem transport, regulation of metabolic enzymes, and biotic and abiotic stress responses. Potassium, nitrogen, and phosphate, are the three main ions consumed by plants and are heavily utilized for fertilization in modern agriculture. (52). The effect of nitrogen and phosphorous on root microbial communities has been characterized (6, 53, 54). However, the role of potassium in bacterial colonization is less well explored. Our analysis suggests that addition of potassium is a simple method to enhance bacillus colonization. Many bacillus species exert positive effects on their plant host by enhancing plant growth and inhibiting pathogens. Our results suggest that adding potassium could enhance the ability of *B. velezensis* to protect seedlings from R. solani infection.

## MATERIALS AND METHODS

### Bacterial strains and growth conditions.

WT B. subtilis NCBI 3610 and AR16 (55) (B. subtilis PY79) were kindly provided by Sigal Ben-Yehuda (Hebrew University); Δ*tagE* (BKK35730), Δ*ktrA* (BKK31090), Δ*ktrC* (BKK14510), and Δ*kimA* (BKK04320) strains (all in B. subtilis 168) were purchased from the Bacillus Genetic Stock Center (http://www.bgsc.org/). Genomic DNA was extracted from AR16 and mutant strains using a Wizard genomic DNA purification kit (Promega) and transformed into B. subtilis NCBI 3610. The media and growth conditions used for DNA transformation of B. subtilis NCBI 3610 are described at http://2013.igem.org/Team:Groningen/protocols/Transformation. The bacteria were cultivated routinely on Luria broth (LB) medium. When needed, the medium was solidified with 1.5% agar. For biofilm formation, bacteria grown overnight were inoculated into MSgg medium and incubated without shaking for 2 days at 30°C as described in reference 20. This medium was also solidified with 1.5% agar.

### Phage strains and infection conditions.

SPO1 (1P4) and SPP1 (1P7) phage were purchased from the Bacillus Genetic Stock Center. Phage lysate was routinely prepared by adding approximately 10^9^ phage to mid-log cells grown in LB medium supplemented with 10 mM MgSO_4_, until the culture was completely cleared. The lysate was then filtered through a 0.22-μm Millipore filter. For lysis dynamics in LB medium (Fig. 2E and Fig. S1B), approximately 10^9^ phage were added to mid-log-phase growing cells at multiplicity of infection (MOI) of 1, and the optical density at 600 nm (OD_600_) was monitored at 30-min intervals. For SPO1 infection in pellicle biofilm (Fig. S3), bacteria grown overnight were inoculated into MSgg medium, and phage was added at a MOI of 1. Bacteria were incubated without shaking for 2 days at 30°C, and the number of cultures with surviving cells was counted by eye. For SPO1 infection in solid biofilm (Fig. 4), bacteria grown overnight were inoculated into MSgg medium supplemented with 10^5^ PFU and solidified with 1.6% agar. A spot of 5 μl from the bacterial culture was inoculated onto the plate and incubated for 2 days at 30°C. Plates were scanned and colony diameter was determined using ImageJ.

### Monitoring bacterial growth and phage infection on plant roots.

Plants were grown on 0.5 MS medium containing 1.1 g Murashige and Skoog basal salts (in 500 ml double-distilled water [ddH2O]), 1% sucrose, 1% agar, and 5 ml (in 500 ml ddH_2_O) morpholineethanesulfonic acid (MES; 50 g/liter, titrated to pH 5.8 with NaOH). Plants were stratified for 2 days in a 4°C dark room and grown vertically for 4 to 10 days under long-day light conditions. Bacteria from fresh colonies were grown in LB medium to an OD_600_ of 1.0 and then diluted 1:100 in 1× phosphate-buffered saline (PBS) for CFU measurements and microscopy, yielding approximately 1 × 10^6^. Phage were added to the 1× PBS at an MOI of 0.1 unless otherwise indicated. Six-day-old seedlings were transferred onto square petri dishes containing 0.5 MS but without sucrose. Two microliters of bacterial dilution was deposited immediately above the root tip and left to dry for 2 min. The square plates were kept in a vertical position during the incubation time at 22°C under long-day light conditions (16 h light/8 h darkness) in a plant growth chamber. For bacterial CFU counting and microscopy, plants were incubated with bacteria for 48 h. Then, the inoculated plant roots were cut and washed three times in sterile water. For CFU counting, the seedlings were transferred to a tube with 1 ml of 1× PBS and vortexed vigorously for 20 s; then, a serial dilution was plated on LB plates. For PFU measurement, roots were treated as described for CFU, but without the washing step.

### Microscopy.

Roots were observed using a Zeiss LSM 880 laser scanning confocal microscope with 20× lens objective. ConA_488_ (Thermo Fisher C11252) staining was performed as described previously (26). Bacteria were observed using a Zeiss LSM 880 laser scanning confocal microscope with a 40× (water immersion) lens objective. Fluorescence intensity was measured using ImageJ.

### DNA sequence analysis.

DNA was extracted from an overnight culture of the mutant bacterial strain along with the WT strain using a DNeasy PowerSoil Pro kit (Qiagen), according to the manufacturer’s instructions. DNA sequencing (DNA-seq) libraries were prepared using a Nextera DNA Flex library preparation kit (Illumina), according to the manufacturer’s instructions. Illumina NextSeq 500 high-output 50-bp single reads were partially assembled into contigs using the SPAdes algorithm (56) with default parameters. The contigs were aligned to the genome of the WT bacteria from our lab stock.

### Data analysis and statistics.

All graphs and statistical tests were performed in Excel. *P* values throughout the paper were determined using Student’s *t* test, except for Fig. S3, where a chi-square test was performed.

### Data availability.

All the data utilized to generate the graph can be found online at https://www.doi.org/10.6084/m9.figshare.14925375. The DNA-seq data set is available in the SRA repository under accession number PRJNA729435.
