# Supplementary figures and images for: Phage-Resistant Bacteria Reveal a Role for Potassium in Root Colonization
Source: mBio. 2021 Aug 17;12(4):e01403-21. doi: 10.1128/mBio.01403-21 (PMC8406301; doi:10.1128/mBio.01403-21)

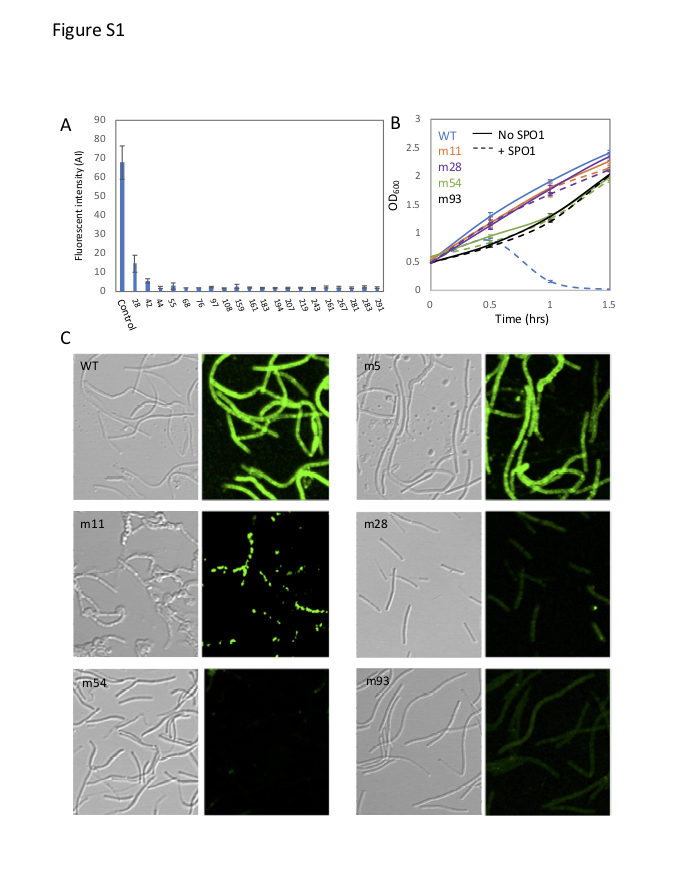

Supplement: FIG S1 [file mbio.01403-21-sf001.tif]

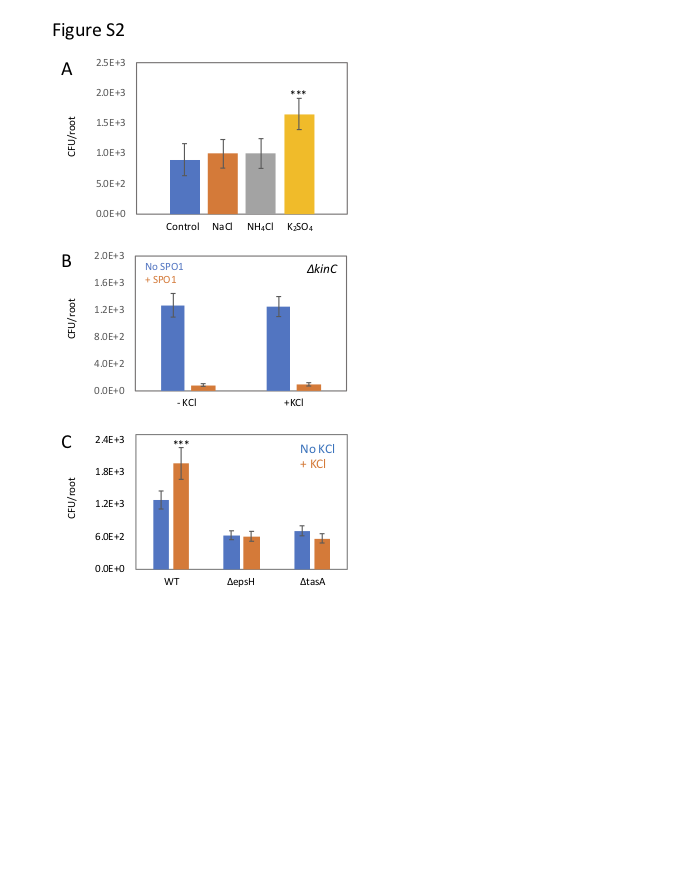

Supplement: FIG S2 [file mbio.01403-21-sf002.tif]

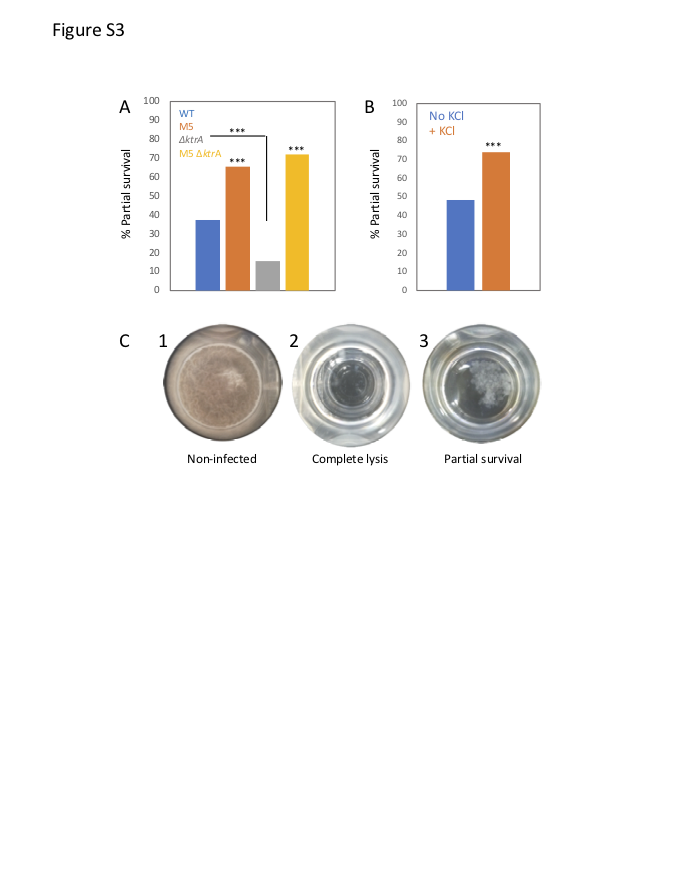

Supplement: FIG S3 [file mbio.01403-21-sf003.tif]
